# *Laimaphelenchus africanus* n. sp. (Tylenchomorpha: Aphelenchoididae) from South Africa, a morphological and molecular phylogenetic study, with an update to the diagnostics of the genus

**DOI:** 10.21307/jofnem-2021-053

**Published:** 2021-05-21

**Authors:** Farahnaz Jahanshahi Afshar, Milad Rashidifard, Joaquín Abolafia, Miloslav Zouhar, Hendrika Fourie, Majid Pedram

**Affiliations:** 1 Iranian Research Institute of Plant Protection, Agricultural Research, Education and Extension Organization (AREEO), Tehran, Iran; 2 Unit for Environmental Sciences and Management, North-West University, Private Bag X6001, Potchefstroom, 2520, South Africa; 3 Departamento de Biología Animal, Biología Vegetal y Ecología, Universidad de Jaén, Campus de las Lagunillas, Avenida de Ben Saprut s/n. 23071, Jaén, Spain; 4 Czech University of Life Sciences, Faculty of Agrobiology, Food and Natural Resources, Department of Plant Protection, Kamycka 129, 165 21, Prague, Czech Republic; 5 Department of Plant Pathology, Faculty of Agriculture, Tarbiat Modares University, Tehran, Iran

**Keywords:** Aphelenchoidea, Compendium, Grouping, LSU rDNA D2-D3, *Pinus pinaster*, Phylogeny, Potchefstroom, Stalk, Taxonomy

## Abstract

A newly recovered population of the genus *Laimaphelenchus* from a dead maritime pine wood sample in Potchefstroom, South Africa, representing a new species, named *L. africanus* n. sp., is herein described and illustrated based on morphological and molecular data. The new species is mainly characterized by the following: 750–987 µm long females; a cephalic region with no disc and six cephalic lobs not divided by ribs; a 10.0–12.5 µm long stylet; four incisures in the lateral field; secretory-excretory pore (SE-pore) at slightly posterior to the nerve ring; vulva with a well-developed anterior flap, vagina with two well-developed sclerotized pieces; post-vulval uterine sac (PUS) 63–125 µm long; tail conical, 30–44 µm long, ventrally curved with a subventral stalk in terminus, lacking tubercles, with six to nine small projections at the tip in scanning electron microscopy (SEM); and rare males with 17 μm long spicules. The new species was morphologically compared to those species of the genus with a stalk in tail terminus, lacking tubercles, a vulval flap and four incisures in the lateral field viz., *L. liaoningensis*, *L. preissii*, *L. simlaensis*, *L. sinensis*, *L. spiﬂatus*, and *L. unituberculus*. Phylogenetically, the new species was placed into the major *Laimaphelenchus* clade using partial large subunit ribosomal DNA (LSU rDNA D2-D3) sequences. An overall literature review corroborated the presence of the stalk (currently with two main groups) at the tail end is the main characteristic trait delimiting the genus. A compendium based on the characters of the stalk, presence/absence of a vulval flap in females and number of the lateral lines was also established.

According to the checklist of Aphelenchoidea (Fuchs, 1937) by Hunt (2008), the genus *Laimaphelenchus* (Fuchs, 1937) belongs to the family Aphelenchoididae (Skarbilovich, 1947) and subfamily Aphelenchoidinae (Skarbilovich, 1947). It was delimited by having four fringed tuberculate processes (three tubercles was reported once, needing further confirmations), or just one tubercle and/or lacking tubercles on the tail tip of both sexes (Hunt, 2008). At the date of Hunt’s checklist, the genus included 13 valid species. The genus was redefined based on the isolation of its species from wood and soil, having four pedunculate tubercles with fringed margins or with a raspberry-shaped appendage on the tail tip, and the possibility of the presence of a vulval flap by females according to Kanzaki and Giblin-Davis (2012). In 2016, *L. heidelbergi* was transferred to the genus *Aphelenchoides* (Fischer, 1894) by Carta et al. (2016), while other possible synonymies were discussed by Pedram et al. (2018). Recently, four species viz., *L. suberensis* (Maleita et al., 2018), *L. liaoningensis* (Song et al., 2020), *L. spiﬂatus* (Gu et al., 2020a), and *L. sinensis* (Gu et al., 2020b) have been added to the genus.

In 2008, when the checklist of Aphelenchoidea was published, molecular data were available only for *L. australis* (Zhao et al., 2006a) and *L. preissii* (Zhao et al., 2006b). Lately, most of the recently described species include molecular data. The molecular data of the type populations improve delimitation of the species, assist in explaining the tentative synonymies (Pedram et al., 2018) and will further clarify the phylogeny of the genus.

The genus is known in South Africa by only one representative, *L. patulus* (Swart, 1997), which has been described based on the traditional criteria. During the present study, a population of the genus, representing an unknown species, was recovered from wood samples of a dead maritime pine tree in Potchefstroom, North-West province, South Africa. Comparisons with all species placed in this genus revealed that it belongs to a new species and is described herein as *Laimaphelenchus africanus* n. sp. This is the second report of a native species of the genus from the African continent. Scanning electron microscopic (SEM) images and molecular sequences are provided for the new species as well as an update to the diagnostics of the genus, focused mainly on the nature of the stalk at the tail end.

## Materials and methods

### Sampling, nematode extraction and morphological observation

Several dead bark and wood samples of coniferous trees (*Pinus pinaster*) were collected in Potchefstroom, South Africa. The samples were cut into smaller pieces for nematode extraction purpose. The tray method of Whitehead and Hemming (1965) was used to extract the nematodes, which were then killed with a hot 4% formaldehyde solution, transferred to anhydrous glycerin using De Grisse (1969) method and mounted on permanent slides. The specimens were examined using a Nikon Eclipse E600 light microscope. Photomicrographs were taken using an Olympus DP72 digital camera attached to an Olympus BX51 microscope equipped with differential interference contrast. Drawings were made using a drawing tube attached to the microscope and were redrawn using the CorelDRAW® software version 2017.

### Scanning electron microscopy

For the scanning electron microscopy, specimens preserved in glycerine were selected for observation under SEM according to the Abolafia’s (2015) protocol. The nematodes were hydrated in distilled water, dehydrated in a graded ethanol-acetone series, critical point dried with liquid carbon dioxide, mounted on SEM stubs, coated with gold, and observed with a Zeiss Merlin microscope (5 kV) (Zeiss, Oberkochen, Germany).

### DNA extraction, PCR, and sequencing

For DNA extraction, two live female individuals of the collected population of *Laimaphelenchus* were isolated, washed using distilled water, observed after being mounted on temporary slides, and photographed. The specimens were then transferred to two individual Eppendorf tubes containing 15 µl ddH_2_O and their respective DNA was extracted using the chelex-100 protocol of Rashidifard et al. (2019). The DNA samples were stored at –20°C until used for amplification. The partial sequences of the large subunit ribosomal DNA (LSU rDNA D2-D3) were ampliﬁed using forward primer D2A (5′–ACAAGTACCGTGAGGGAAAGT–3′) and reverse primer D3B (5′–TCGGAAGGAACCAGCTACTA–3′) (Nunn, 1992). The polymerase chain reaction (PCR) was performed in the same conditions describe by Pedram (2019). The newly obtained LSU D2-D3 sequences were deposited into the GenBank database under the accession numbers MW507183 and MW507184.

### Phylogenetic analyses

The raw file of the newly generated partial sequences of LSU rDNA of *Laimaphelenchus africanus* n. sp. were manually checked, edited, and compared with those of the relevant sequences available in the GenBank database using the BLAST homology search program. Sequences of several representatives of the aphelenchoidids were selected for LSU phylogeny. The multiple alignment of 87 selected sequences was conducted using MUSCLE (Edgar, 2004) as implemented in MEGA6 (Tamura et al., 2013). The resultant alignment was edited manually. The best-ﬁtting substitution model was selected using the Akaike information criterion (AIC) by using PAUP*/MrModeltest v2.2 (Nylander, 2004). A general time reversible model, with proportion of invariable sites and a gamma distribution (GTR + I + G) was selected for the phylogenetic analysis. Bayesian inference (BI) was performed using MrBayes v3.1.2 (Ronquist and Huelsenbeck, 2003) and a random starting tree, running the chains for 5 × 10^6^ generations. After discarding burn-in samples, the remaining samples were retained for further analyses. The Markov chain Monte Carlo (MCMC) method within a Bayesian framework was used to estimate the posterior probabilities of the phylogenetic trees (Larget and Simon, 1999) using the 50% majority rule. The resultant phylogenetic tree was visualized with Dendroscope V.3.2.8 (Huson and Scornavacca, 2012) and drawn in CorelDRAW® software version 2017.

## Results

### Systematics

**Figure 1:**
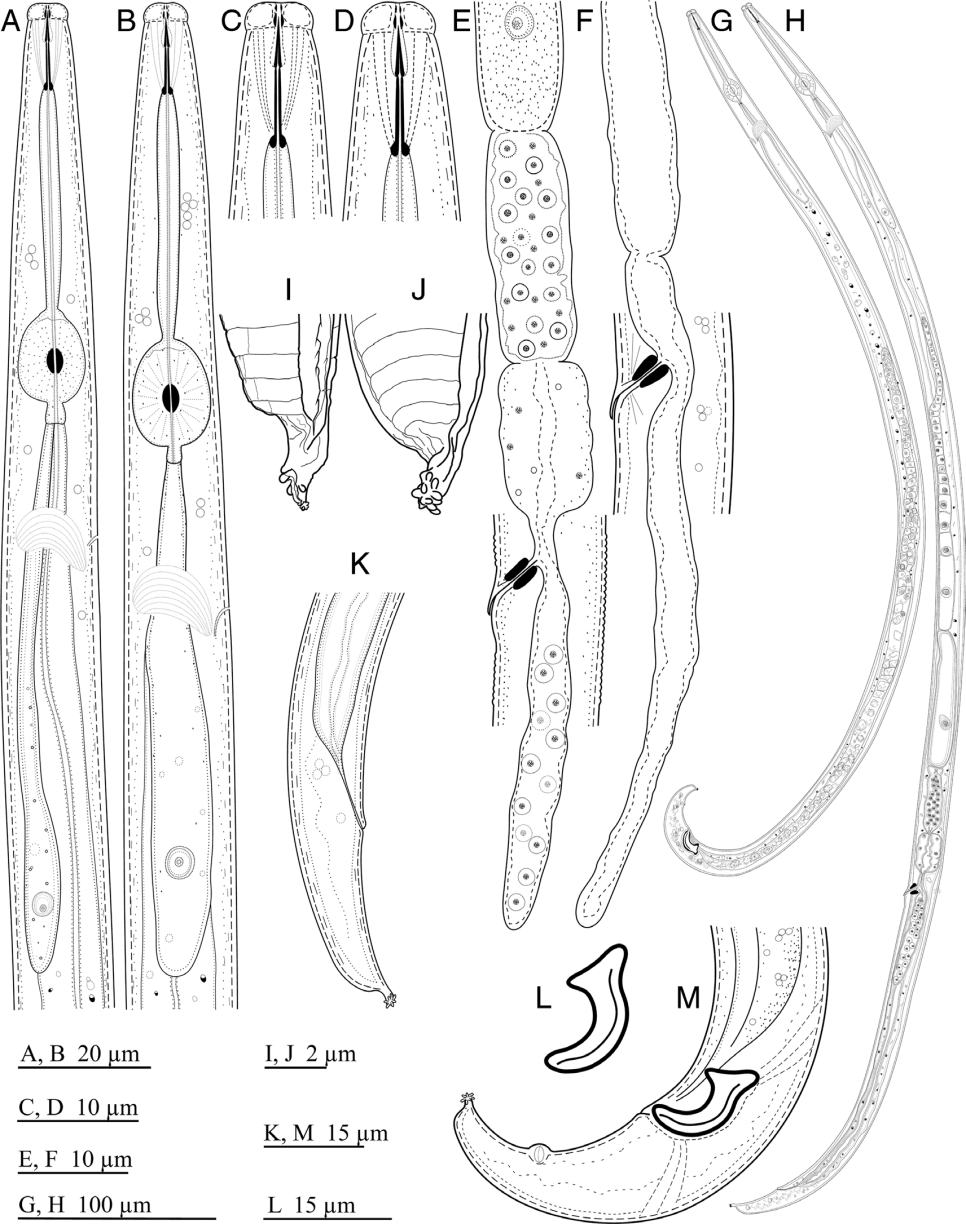
Line drawings of *Laimaphelenchus africanus* n. sp. (B, D, G, L, M: Male; A, C, E, F, H-K: Female). (A and B): Pharynx; (C and D): Anterior end; (E and F): Part of reproductive system; (G and H): Total body; (I and J): Details of the stalk at the tail tip; (K and M): Posterior body region; (L): Spicule.

**Figure 2:**
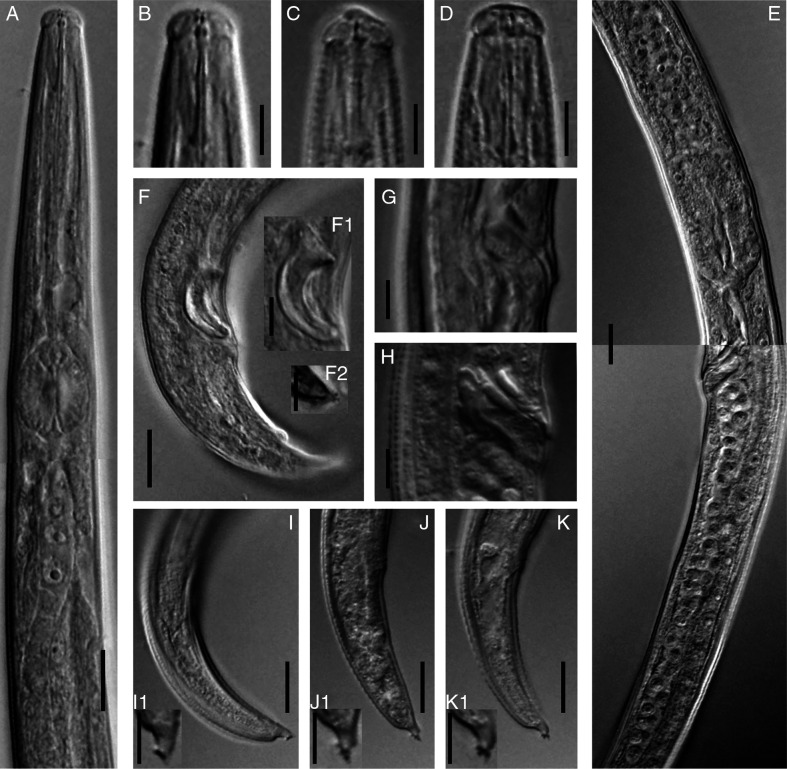
Light photomicrographs of *Laimaphelenchus africanus* n. sp. (A-C, E, G-K, female; D, F, F1, F2, male). (A): Part of pharynx; (B-D): Anterior end; (E): Part of reproductive system; (G and H): Vulva; (F, I-K): Posterior body region; (F1): Spicule; (F2, I1-K1): Details of the stalk at the tail tip. (Scale bars: B-D, F1, F2, G, H, I1-K1 = 5 µm; A, E, F, I-K = 10 µm).

**Figure 3:**
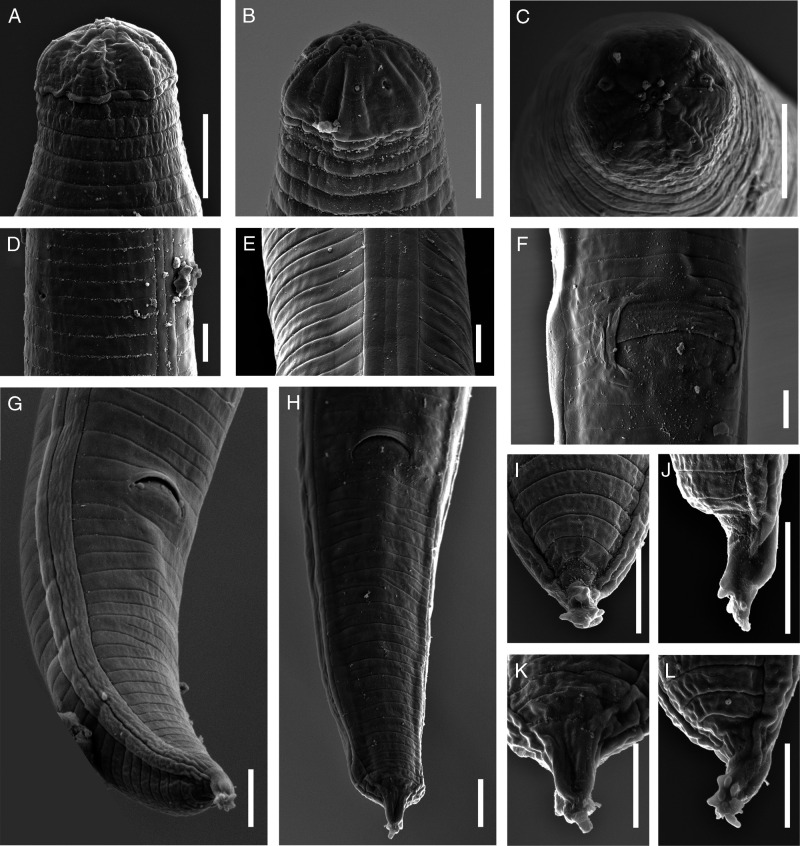
Scanning electronic microscopy of *Laimaphelenchus africanus* n. sp., female. (A-C): Details of anterior end; (D): Secretory-excretory pore; (E): Lateral field; (F): Vulva in ventral view; (G and H): Tail in ventro-lateral and ventral views; (I-L): Details of the stalk at the tail tip. (Scale bars = 3 µm)

*Laimaphelenchus africanus* n. sp. (Figs 1–3).

### Measurements

Measurements of the new species are given in Table 1.

**Table 1.** Morphometric characteristics of *Laimaphelenchus africanus* n. sp.

Characteristics	Holotype female	Paratype females	Paratype male
n	‒	21	1
L (micron)	916	898 ± 67 (750–987)	696
a	48.2	42.6 ± 3.0 (37.0–48.2)	43.5
b	11.2	11.6 ± 0.6 (10.8–12.7)	10.4
b′	4.4	4.7 ± 0.4 (4.1–5.7)	4.9
c	22.3	24.3 ± 2.3 (20.8–30.8)	17.8
c′	3.4	3.2 ± 0.4 (2.5–3.8)	2.4
V or T %	71.2	70.0 ± 1.1 (67.1–71.9)	58.2
Cephalic region width	7	6.8 ± 0.4 (6–7)	6.5
Cephalic region height	2.5	2.8 ± 0.5 (2–4)	3
Stylet length	12.5	11.8 ± 0.6 (10.0–12.5)	12
Conus length	6	4.9 ± 0.5 (4–6)	5
m	48	41.7 ± 3.2 (34.8–50.0)	41.7
Anterior end to valves of median bulb	63	62.0 ± 4.1 (53–67)	56
MB	76.8	80.0 ± 2.5 (76.5–84.4)	83.6
Anterior end to nerve ring	100	97.0 ± 6.8 (80–106)	82
Anterior end to pharyngeal intestinal junction	82	77.0 ± 5.5 (65–85)	67
Anterior end to posterior of pharyngeal glands	206	192 ± 17.6 (150–217)	143
Overlap	124	115.7 ± 14.2 (83–141)	76
Median bulb width	15	12.0 ± 1.3 (10–15)	10.5
Median bulb length	17	16.0 ± 1.3 (13–18)	15
Diam. at median bulb	15	16.0 ± 0.9 (14–17)	‒
Max. diam.	19	21.0 ± 2.1 (16–25)	16
Median bulb length/diam. ratio	1.1	1.4 ± 0.1 (1.1–1.6)	1.4
Anterior to SE-pore	102	100 ± 7.4 (87–112)	86
Anterior end-vulva	652	628 ± 50.4 (520–700)	‒
Post-vulval uterine sac (PUS)	100	91.0 ± 14.2 (63–125)	‒
Vulva to anus distance	308	238 ± 73.4 (139–388)	‒
PUS/vulva to anus (%)	32.5	41.3 ± 11.6 (23.2–63.1)	‒
PUS/L (%)	10.9	10.2 ± 1.3 (7.6–12.7)	‒
Diam. at anus or cloaca	12	12.0 ± 1.1 (9–13)	16
Tail	41	37.0 ± 3.6 (30–44)	39
Spicules (arc)	‒	‒	17
Spicules (chord)			16.6
Calamus	‒	‒	7
Spicules width	‒	‒	5

### Female

Body slender, slightly arcuate ventrally when heat relaxed. Cuticle with fine transverse annulations, 1.0–1.5 µm wide at mid-body according to SEM. Lateral field marked by four incisures, making three bands, the inner one narrower than the two outer ones as visible using SEM (Fig. 3E). Cephalic region rounded, offset by a shallow constriction under light microscopy (LM), 2–4 µm high and 6–7 µm wide, without a labial disc and with six equally sized lips not separated by ribs (visible under SEM). Cephalic papillae four at mid-position of lip region height, labial papillae six surrounding the oral aperture (Fig. 3C). Amphidial openings pore-like, located at mid-position of lateral lips height, slightly dorsally shifted. Stylet slender, anterior conical part about ½ of the total, shaft with three small swellings (Fig. 2C). Pharynx with procorpus cylindrical, 32–43 µm long, median bulb (metacorpus) oval, 13–18 µm long, 10–15 µm wide, with a centrally located valve, 53–67 µm from the anterior end. The pharyngo-intestinal junction (cardia) subcylindrical often with distanced lumen. The dorsal pharyngeal gland well-developed, slender, with three visible nuclei, overlapping intestinal dorsally for 83–141 µm long (Fig. 1A). Nerve ring at 1.2–1.9 times maximum body width posterior to the median bulb, 80–106 µm from the anterior end. Secretory-excretory pore (SE-pore) almost opposite to the nerve ring. Hemizonid not observed. Genital tract mono-prodelphic, ovary outstretched with oocytes in a single row, oviduct short, spermatheca oval to oblong filled with amoeboid (spheroid to oval) sperm cells, crustaformeria with no clearly seen cells, uterus with a wide lumen, vagina directed anteriorly, the sclerotized pieces large, vulva a transverse slit with a well-developed vulval flap overlapping the posterior lip, post-vulval uterine sac (PUS) 63–125 µm long, occupying 3.2–5.3 times vulval body diameter or 23.2–63.1% of the distance from vulva to anus, containing sperm in some individuals. Anus distinct, well developed. Tail conical, ventrally curved, dorsally convex, with a subventral stalk in terminus, lacking tubercles, having six to nine small projections at the tip of the stalk (Fig. 3I–L).

### Male

Rare. Only one specimen was recovered. Body slender, similar to that of the females except genital system and posterior body end more ventrally bent. Testis single, outstretched, developing spermatocytes in a single column. Spicules curved, 17 μm long along arc line, capitulum without clear depression in middle, blade (calamus-lamina complex) smoothly ventrally arcuate, condylus bluntly rounded, rostrum triangle-shaped with blunt tip and distal end of spicules bluntly rounded. Only one pair of subventral papillae observed (P3), located at middle of the tail. Bursa absent. Tail curved ventrally, its terminus similar to that of females.

### Type host and locality

During August 2019, bark and wood were sampled from maritime pine trees (*Pinus pinaster*) showing gradual decline since 2010 in Potchefstroom, North West province, South Africa (26°42′18.0″ S, 27°07′05.4″ E; elevation 1,353 m.a.s.l.).

### Type specimens

The holotype female (accession number: 51317) and four paratype females (accession number: 51318) were deposited in the National Collection of Nematodes (NCN), ARC-PPRI, Pretoria, South Africa. 14 paratype females and paratype male were deposited in the Nematode Collection of Faculty of Agriculture, Tarbiat Modares University, Tehran, Iran. Three paratype females were deposited in the WaNeCo collection, Wageningen, The Netherlands (http://www.waneco.eu/).

### Etymology

The speciﬁc epithet refers to the name of its native continent.

### Differential diagnosis

*Laimaphelenchus africanus* n. sp. is characterized by 898 (750–987) µm long females, cephalic region with six lobs not divided by ribs and no disc, 11.8 (10.0– 12.5) μm long stylet, four incisures in the lateral field, SE-pore slightly posterior to the nerve ring, vulva with a well-developed anterior flap, vagina with two well-developed sclerotized pieces, 91 (63–125) µm long PUS, tail 37 (30–44) µm long with a subventral stalk in terminus lacking tubercles but having six to nine small projections at the tip in SEM and rare male with 17 μm long spicules.

By having a tail with a subventral stalk lacking tubercles but with several small projections at the tip, vulval flap in females, and the lateral field with four incisures, the new species resembles five known species of the genus namely: *L. preissii*, *L. simlaensis* (Negi et al., 2009), *L. sinensis*, *L. spiﬂatus*, *L. liaoningensis*, and *L. unituberculus* (Bajaj and Walia, 2000), but can morphologically be separated from them as follows:

It is distinguished from *L. preissii* by shorter female body length (898 (750–987) vs 1,185 (1,007–1,386) µm), male (696 vs 1,088 (1,000–1,218) µm), female stylet (11.8 (10.0–12.5) vs 14 (12–15) µm) and spicules (17 vs 22–28 µm).

It is distinguished from *L. sinensis*, by slightly shorter female body length (898 (750–987) vs 968 (914–1,064) µm), larger vulval flap (vs smaller) and longer (17 vs 14.0 (13.2–15.0) µm) and differently shaped spicules (curved vs not).

It is distinguished from *L. simlaensis* by shorter distance from anterior end to valve of median bulb (61.7 (53–67) vs 70–80 µm), sclerotized vagina (vs not), the nature of the stalk at the tail end of female (having 6–9 projections vs 3–5 finger-like fine processes), and spicules morphology (curved with smaller condylus and blunt rostrum vs slightly curved with large condylus and rostrum with sharp tip).

It is distinguished from *L. unituberculus* by more posterior SE-pore (100 (87–112) vs 82–85 µm) and nerve ring (1.2–1.9 vs one body width posterior to median bulb), the nature of the stalk at the tail end of female (having 6–9 projections vs ending to a saucer-like surface with bristle-like appendages at around, after its original drawings) and longer (17 vs 14–15 µm) and differently shaped spicules (curved vs rose-thorn).

It is distinguished from *L. spiﬂatus* by shorter female body length (898 (750–987) vs 1,150 (976–1,437) μm), male (696 vs 1,092 (905–1,235) µm), female tail (37.1 (30–44) μm, c′ = 3.2 (2.5–3.8) vs 55 (48–66) μm, c′ = 4.2 (3.8–4.9)), the nature of the stalk at the tail end of female (having 6–9 projections vs 8–12 ﬁnger-like projections), shorter (17 vs 27.3 (23.4–28.8) μm) and differently shaped spicules distal end (rounded vs truncate).

It is distinguished from *L. liaoningensis* by shorter female body length (898 (750–987) vs 1,462 (1,252–1,722) μm), male (696 vs 1,206 (972–1,383) µm), female tail (37.1 (30–44) μm, c′ = 3.2 (2.5–3.8) vs 62 (53–70) μm, c′ = 3.6 (3.1–4.1)), the nature of the stalk at the tail end of female (having 6–9 projections vs with two tubercles with four to six finger-like protrusions) and shorter (17 vs 28 (24–30) μm) spicules.

### Molecular profile and phylogenetic status

**Figure 4:**
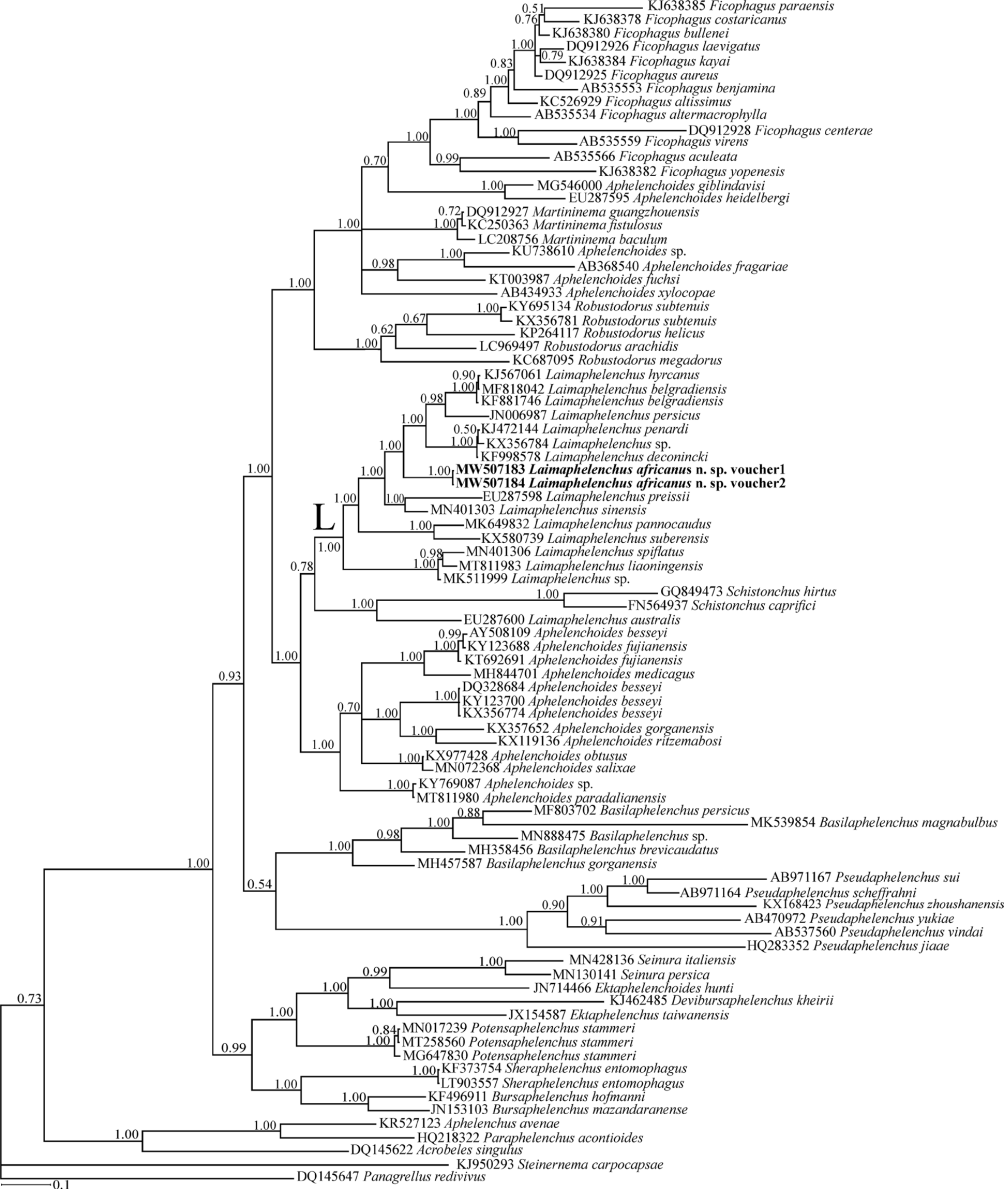
Bayesian 50% majority rule consensus tree of *Laimaphelenchus africanus* n. sp. based on LSU rDNA D2-D3 sequences under GTR + I + G model. Bayesian posterior probability values more than 0.50 are given for appropriate clades. The new sequences are indicated in bold.

**Figure 5:**
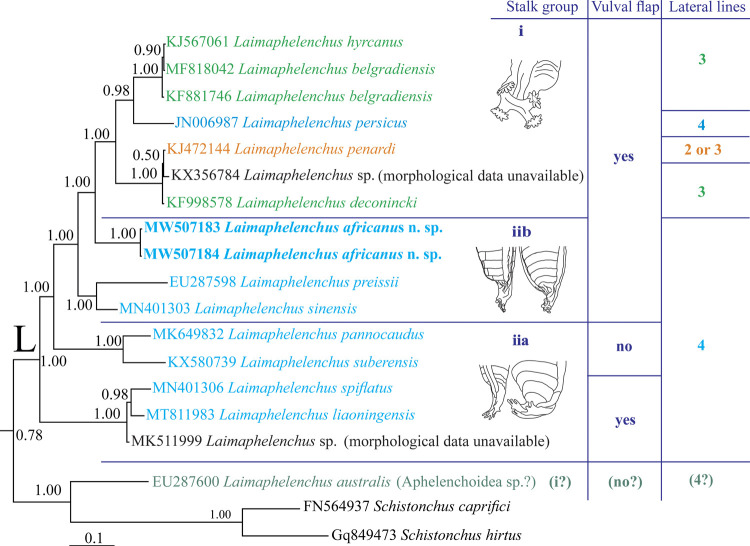
The close-up view (pruned tree) showing the clade L in the original Bayesian 50% majority rule consensus tree of *Laimaphelenchus africanus* n. sp. based on LSU rDNA D2-D3 sequences under GTR + I + G model. Data on stalk type, vulval flap status and lateral lines number for the currently sequenced species are given in right.

The two newly generated identically aligned LSU D2-D3 sequences of *Laimaphelenchus africanus* n. sp. (MW507183 and MW507184) were 669 and 709 nt long. The BLAST search using the longer sequence revealed the identity of this new species with currently available sequences deposited into the database, were less than 90%. A total number of 82 LSU sequences of aphelenchoidids (including two newly generated sequences of the new species), with five sequences of aphelenchids as well as classic rhabditids as outgroups, were used for inferring the LSU phylogeny. The dataset included 927 characters of which 720 character were variable. The Bayesian phylogenetic tree inferred from this dataset is presented in Fig. 4. The currently sequenced *Laimaphelenchus* spp. for their LSU D2-D3, except *L. australis* (see Discussion section), formed a maximally supported clade in this tree (the clade L) and *L. africanus* n. sp. appeared as an independent lineage in this clade. The pruned smaller tree as represented in Fig. 5 shows the clade L and data on stalk type, vulval flap status and lateral lines number for the currently sequenced species.

## Discussion

The present study aimed to identify the native species of *Laimaphelenchus* occurring in South Africa. The newly discovered species increases our knowledge on representatives of the genus in the country. Previously, only one species, *L. patulus*, had been reported from the region (Swart, 1997).

The genus was characterized by Hunt (1993) as follows: “tail tip bearing four pedunculate tubercles with fringed margins. A vulval flap, formed by the posterior extension of the anterior lip, may be present.” The diagnostics of the genus were updated after adding another species to the genus, namely *L. unituberculus*, a species with just one tubercle. Later, other species having a similar structure at the tail end were added. In his checklist of Aphelenchoidea, Hunt (2008) stated “clearly, the value of such ‘diagnostic’ morphological characters will be better resolved once molecular characterization is more widely applied.” It seems, based on available data, that having a stalk at the tail end of females and disregarding the type of its differentiation at tip, well delimits the genus. The stalk at the tail tip could be divided into two major types as follows: (i) the stalk has four (rarely three, see Table 2) tubercles, each tubercle having a saucer-like tip including fringed finger-like appendages, (ii) the stalk lacks tubercles, but has flat fused stacked structures with finger-like appendages (iia) or projections (iib), or a warty surface at tip (iic) (see Table 2 for the species belonging to each group). The latter form, however, should not be misinterpreted as a warty mucro, a differentiation at the tail tip of some recently described species of *Aphelenchoides*, e.g. *A. giblindavisi* (Aliramaji et al., 2018) and *A. hamospiculatus* (Mortazavi and Pedram, 2020).As already stated, the tentative/possible synonymies for some species of the genus were recently addressed by Pedram et al. (2018). During the present study, a detailed examination of the original descriptions of *L. patulus* and *L. australis* suggested that they could belong to the same species, as the used traits to differentiate the latter species from the former look insufficient (the status of the lateral line in the former species needs further studies). The given range for the ‘V’ of *L. australis* (50.0–83.9) does also need a revision. The lack of sequences for type populations of two aforementioned species, however, does not allow decisive judgment on their status for which the same situations were reported for the following two species: *L. deconincki* (Elmiligy and Geraert, 1972) and *L. penardi* (Filipjev and Schuurmans Stekhoven, 1941; Steiner, 1914) (Pedram et al., 2018). The two species *L. liaoningensis* and *L. spiflatus* seem also to belong to the same species, and the few differences at the 5ʹ end of the D2–D3 sequences of these species while aligning, could be due to the poor quality of one of them. A bursa that is reported for *L. preissii* and *L. pensobrinus* (Massey, 1966), however, seems to be the result of optical illusion, and an erroneous interpretation of the flattened posterior body region, or the slightly raised lateral field at the posterior body end. The description of the male of the latter species was also improved by Massey (1974) since the bursa of males were excluded.

**Table 2.**
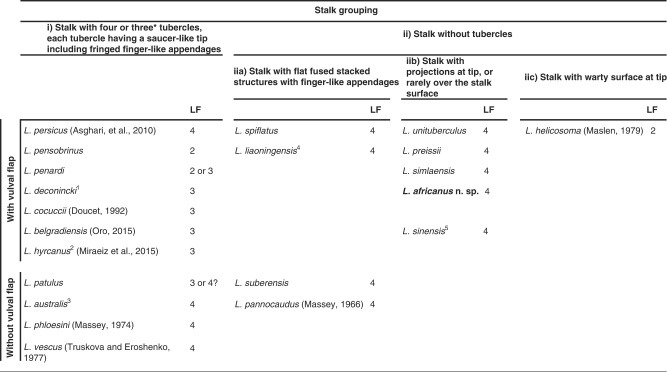
Compendium of *Laimaphelenchus* spp. arranged based on stalk characters, presence/absence of vulval flap and lateral lines number.

Recently, molecular methods have been extensively utilized for species characterization since molecular data were generated for all those recently described. The current study represents the latest molecular study of the genus, and shows it could be monophyletic based upon LSU data. The exact identity of the accession number EU287600 assigned to *L. australis*, occupying a placement outside the clade of *Laimaphelenchus* spp. is not clear, as its morphological data are not available, and it is better to be renamed as Aphelenchoidea sp. Resequencing of the type population of *L. australis* would clarify the status of the species and the aforementioned sequence. One obstacle in the molecular phylogeny of the genus is the identity of the species deposited into GenBank under the name *Aphelenchoides* sp., most of which lack morphological data. Misidentification of the generic status of the sequenced specimens due to typological similarity of *Laimaphelenchus* and *Aphelenchoides* is a possible scenario. The present LSU phylogeny including the new species; and the resolved topology, is an update to the recently resolved phylogeny by Gu et al. (2020a, b), showing that a new sequence could affect the cladogenesis events by yielding better resolution of the relationships and improving the clade supports. It is yet to be elucidated how including new sequences will update the currently available SSU phylogenies of the genus (e.g. Gu et al. 2020a, b). During the present study, our efforts to amplify and sequence the SSU locus of the new species failed. Interestingly, adding further molecular data would likely result in better clarity in the phylogeny of the genus, a similar case was reported for *Robustodoru*s Andrássy, 2007 (Aliramaji et al., 2018; Kanzaki et al., 2018), *Cryptaphelenchus* Fuchs, 1937 (e.g. Pedram et al., 2020) and *Seinura* Fuchs, 1931 (e.g. Gu et al., 2020a, b); corroborating that further molecular data improved and elucidated their phylogeny status. Despite the fact that based on the current data the three aforementioned genera might be monophyletic, the methodology of inferring the phylogenies (e.g. aligning, postediting methods or the used methods) are other factors that may influence the resolved topologies.

## Conclusion

The taxonomy of *Laimaphelenchus* has attracted attention in recent years. After recent studies, tentative synonymies are imagined for the currently valid species. Here we propose that adding of the new species to the genus to be done more prudently, by using remarkable/significant morphological and morphometric data, and when possible, by examining type materials of close species. The molecular data should also be included while establishing new species, and a future sequencing of topotypes of currently known species will help to better clarify their status.
